# Refinement of transvaginal ovum pick-up in sows increases oocyte recovery and supports in vitro embryo production

**DOI:** 10.1186/s13028-026-00865-6

**Published:** 2026-04-16

**Authors:** Aslak Oltedal, Reina Jochems, Ann Helen Gaustad, Olli Peltoniemi, Stefan Björkman, Marianne Oropeza-Moe

**Affiliations:** 1 Department of Production Animal Clinical Sciences, Norwegian University of Life Sciences, Sandnes, 4325 Norway; 2 Norsvin SA, Hamar, 2317 Norway; 3 Department of Production Animal Medicine, Faculty of Veterinary Medicine, University of Helsinki, Helsinki, 00014 Finland

**Keywords:** Aspiration pressure, Epidural, In vitro, In vivo, Embryo, Porcine, Sedation

## Abstract

**Background:**

Efficient recovery of oocytes from sows may facilitate embryo production and increase the number of offspring obtained from breeding sows with desired traits. However, robust protocols for consistent oocyte retrieval in sows are still lacking. The aim of this field study was to evaluate modifications of a transvaginal OPU protocol in multiparous sows, including the use of anaesthetics and analgesics, different aspiration flow rates, and hormonal treatments. Furthermore, the effect of partial in vivo oocyte maturation prior to in vitro embryo production (IVEP) was assessed. Thirty-two hybrid sows (parity 5–7) were included in three experiments. In Experiment 1, short-term altrenogest treatment combined with equine chorionic gonadotropin (eCG) and human chorionic gonadotropin (hCG) was used to attempt synchronization of follicular development. In Experiment 2, the effects of superstimulation (1000 or 1500 IU eCG), day post-weaning, and two aspiration flow rates of 24 and 30 mL of water per minute were evaluated. In Experiment 3, OPU-derived oocytes collected 10–14 h or 34–36 h after hCG administration were subjected to IVEP to assess the effect of partial in vivo maturation.

**Results:**

In Experiment 1, altrenogest-based synchronization was associated with low oocyte recovery in several animals. In Experiment 2, a mean of 30.3 ± 11.0 oocytes per OPU session was recovered from 54.8 ± 10.6 aspirated follicles, corresponding to a recovery rate of 55.3%. No significant differences were detected between the two aspiration flow rates or between superstimulation treatments. In Experiment 3, partially in vivo matured oocytes resulted in a 33% blastocyst rate, compared with 6% following full in vitro maturation.

**Conclusions:**

These results demonstrate that transvaginal OPU in sedated, epidurally treated sows can yield substantial numbers of oocytes under field conditions. Partial in vivo maturation prior to IVEP was associated with improved blastocyst rates. Further controlled studies are required to optimize synchronization protocols and to improve repeatability of oocyte retrieval and IVEP.

## Background

Embryo transfer (ET) has potential applications in the global market of pig genetics, providing a cost-efficient method of genetic exchange while mitigating biosecurity and welfare concerns associated with the transport of live pigs [1, 2]. Transvaginal ovum pick-up (OPU) holds the potential to enable indispensable research for future commercial breeding programs using advanced assisted reproductive technologies including embryo transfer protocols in pigs, as well as in the development of human and animal biomedical applications. This technique has been widely adopted for oocyte retrieval in the commercial breeding of horses and cattle [3], and in assisted human reproduction [4]. Since the first successful transvaginal OPU in cattle in 1988 [5], there have been continuous improvements of the technique, leading to a robust system for embryo production utilized by commercial breeding programs worldwide [6]. The transvaginal technique offers a less invasive alternative to the laparoscopic approach for OPU [7]. However, initial reports of porcine transvaginal OPU highlighted several challenges, such as animal immobilization and transrectal manipulation in the relatively small pelvic cavity of a non-epidurally blocked sow [8, 9]. Despite these challenges, recent advancements have demonstrated the possibility for repeated oocyte retrievals from high-yielding sows [10–12]. From a welfare perspective, systematic assessment of anaesthetic and analgesic strategies is necessary to minimize stress and discomfort associated with transvaginal OPU in sows. Around 50 follicles may be available for aspiration during the early follicular phase in hyperprolific sows [13, 14], but to date, no robust protocols including the use of anaesthetics and analgesics for retrieval of high oocyte numbers have been published.

Studies investigating laparoscopic OPU in gilts [15], transvaginal ultrasound-guided OPU in sows [12, 16], and aspiration from ovaries collected post-mortem [17, 18] have aimed to identify the optimal aspiration pressure to maximize the retrieval rate of developmentally competent oocytes per session in pigs. Mechanical forces generated during follicular aspiration can influence cumulus oocyte complexes (COCs) integrity and subsequent developmental competence [15]. Bols et al. suggested that expressing the pressure exerted through the needle tip as flow rate (mL fluid/min) through the needle, rather than as mmHg aspiration pressure, would better estimate the effect on the oocyte and allow for better comparison of results [19]. However, many studies do not specify the flow rate, and thus mmHg is frequently used as a measure for aspiration pressure, which complicates the comparison of different studies. What should also be mentioned is that tubing diameters and dimensions as well as temperature conditions and needle types must be stated, to enable others to repeat OPU studies.

In modern pig production, hormonal synchronization of sows with equine chorionic gonadotropin (eCG) and human chorionic gonadotropin (hCG) has been widely applied for both control and timing of estrus and ovulation. Previous studies have also highlighted the possibility of increasing the developmental competence of porcine oocytes via hormonal stimulation of donors before OPU [20]. The LH surge is important for inducing cumulus expansion and germinal vesicle breakdown [21]. Equine Chorionic Gonadotropin (eCG) is unique among mammalian Luteinizing Hormones (LHs) as it can simulate the effects of both LH and FSH [22]. This hormone been frequently used for superstimulation protocols in sows [23], and has proven effective in increasing embryo production [24]. The combination of eCG and human chorionic gonadotrophin (hCG) has been demonstrated to effectively synchronize the in vivo maturation of porcine oocytes [20]. However, issues like polyspermy and incomplete cytoplasmic maturation of the oocyte are well-known challenges with porcine in vitro embryo production [14, 25]. The method of fixed-time in vivo maturation circumvents the challenges associated with replicating in vivo conditions during in vitro maturation (IVM) of oocytes and thus improve the developmental competence of the oocytes. Hence, it is the preferred approach in human IVF procedures for embryo transfer purposes [4]. The efficiency of in vivo maturation versus in vitro maturation, measured in terms of the number of embryos (blastocysts with blastocoel) produced per session, has not yet been evaluated in pigs.

The minimally invasive OPU technique enables repeated on-farm retrieval of oocytes from genetically valuable donors. Initial experiences with repeated OPU procedures on sows are encouraging with respect to the maintenance of reproductive health [11]. In this study, we evaluated modifications of a transvaginal OPU protocol in multiparous sows, including the use of anaesthetics and analgesics, different aspiration flow rates, and hormonal treatments. Furthermore, the effect of partial in vivo oocyte maturation prior to in vitro embryo production was assessed.

## Methods

The main objective of this study was to maximize the number of developmentally competent oocytes retrieved from follicles by incorporating sedation and epidural anaesthesia into our established protocol for transvaginal OPU. The secondary objectives included testing of synchronization and postponing of follicle maturation through altrenogest administration (Experiment 1), testing protocols for superstimulation (Experiments 1 and 2), and evaluating the effect of two different flow rates (Experiments 1 and 2) on the quality of retrieved oocytes. Lastly, the study assessed the impact of partial oocyte maturation in vivo on the efficiency of IVEP (Experiment 3).

The Experiments were approved by the Norwegian Animal Research Authority (permission ID 30400) regarding the Norwegian regulation on animal experimentation, as a field study (FOR-2015-06-18-761).

### Animals and management

The Experiments included a total of 32 hybrid sows (Topigs Norsvin TN70 or Landrace; parity 5–7; age 33.6 ± 2.1 months; body condition score (BCS) 3.1 ± 0.3; lactation length 33.9 ± 1.2 days) housed in a conventional commercial sow pool [26]. All sows had ad lib access to a full-concentrate diet (Avlsfôr Netto, 9.7 MJ NE/kg, 15% crude protein, 7.6 g digestible lysine/kg, Fiskå Mølle AS, Norway) from the day of weaning and throughout the experimental period. Continuous access to water was provided through nipple drinkers. The sows included in Experiments 1 and 2 were selected at weaning and placed together with other sows in the insemination compartment upon arrival at the sow pool. Sows for Experiment 3 were accommodated in a distinct pen following arrival.

### Follicular aspiration and quality grading of retrieved oocytes

The same veterinarian (operator) conducted all OPU sessions in the Experiments 1, 2 and 3. A SonoSite M-turbo ultrasound machine with a C11x ultrasound transducer (Fujifilm SonoSite BV, Amsterdam, The Netherlands) in a customized OPU device [11] for sows was used for transvaginal imaging and needle guidance. The OPU device was designed to hold a commercial stainless-steel tube with a needle adapter (Minitübe, Tiefenbach, Germany, Ref.: 23360/0610), the 200 cm silicone tubing with an inner diameter of 2.0 mm (Minitübe, Tiefenbach, Germany, Ref.: 23360/1000) and an 18 G, 70 mm hypodermic needle to an aspiration pump (Minitübe, Tiefenbach, Germany, Ref.: 23362/0000) for controlled collection of follicular fluid into 50 mL Falcon tubes. A custom-made metal cone was fitted into the OPU device with the intention of stabilizing the needle and thereby enabling more precise follicle punction.

All sows were treated with 0.4 mg/kg of meloxicam (Melovem 20 mg/mL, Dopharma Raamsdonksveer, Holland) i.m. to manage post-procedural pain before administrating anaesthetics and other analgesics. In each treatment group, sows were sedated immediately before the procedure with an injection of 0.16 mg/kg detomidine (Domosedan vet. 10 mg/mL, Orion Pharma, Espoo, Finland) and 0.16 mg/kg butorphanol i.m. (Butorgesic vet. 10 mg/mL, CP-Pharma, Burgdorf, Germany). The sows were restrained and lifted approximately 1.5 m above the floor using a claw-trimming chute (Piggy Trim, Bovi Hoof Care, Denmark). If the sows did not reach the desired depth of anaesthesia (depressed consciousness, not easily aroused), additional boli of 0.04–0.08 mg/kg of detomidine and 0.04–0.08 mg/kg of butorphanol were administered i.m. or i.v. in the auricular vein. For epidural anaesthesia, 20 mg of procaine (Procamidor vet. 20 mg/mL, VetViva Richter, Wels, Austria) was administered after preparing the area between both tuber sacrale by clipping of bristles and disinfection of the injection site. This epidural dosage was chosen to obtain reduced discomfort during rectal palpation and OPU, and at the same time with the aim of maintaining hindlimb mobility during the procedure. Local anaesthesia was applied at the puncture site for epidural anaesthesia by injection of 100 mg of procaine s.c. and i.m. before introducing the epidural needle. The accurate positioning of the needle was ensured by identifying the anatomical landmarks bilaterally of tuber sacrale (dorsal nodes) and inserting the needle guide 1–2 cm behind a line between the landmarks. Signs including the palpable penetration of the ligamentum flavum and a decreased resistance to injection confirmed epidural position of the needle [27]. The rectum was emptied, and the perineal area was cleaned using paper towels, wet wipes, and a 70% ethanol disinfectant spray. Ultrasound gel was applied to the probe before a disposable cover (Mediware, Wesel, Germany, Ref.: H5 1320) was pulled over the complete device. The OPU-device was inserted in the vagina by the operator and held in position close to the external orifice of the cervix by an assistant. The ovaries were aligned with the central axis of the ultrasound transducer beam by transrectal translocation. All accessible follicles were punctured with the needle and aspirated. If subsequent follicles could not be positioned for aspiration within few seconds after the last retrieval, flushing of the needle tubing with heparinized (10 IU heparin/mL) TL-HEPES-PVA (Exp 3) or BoviFlush embryo recovery medium (Exp 1 and 2, Minitube, Ref.: 19982/6012) was carried out to prevent clot formation inside the needle or the tube. The sows were monitored closely by trained personnel until they regained full consciousness.

The follicular fluid from the Falcon tubes was filtered and washed using TL-HEPES-PVA supplemented with 10 IU heparin/mL through an embryo filter (Minitübe, Tiefenbach, Germany, Ref.: 19010/6000). The filtered content was then transferred to a petri dish and examined under a stereomicroscope with up to 50x magnification (ZE.1624, Euromex, VB Duiven, Holland) for isolation and grading of the cumulus-oocyte complexes (COCs). Oocyte quality was evaluated and classified according to the grading scale published by Suzuki et al. [28]. We included expanded COCs as a distinct grade for this study. Oocytes were graded from A to E, according to the following criteria: A: Oocyte with several compact cumulus cell layers; B: Intact corona radiata with at least one additional cumulus cell layer; C: Oocyte with incomplete cumulus cell layers or partially denuded oocyte; D: Completely denuded or degenerated oocyte; E: Oocyte with expanded cumulus oophorus.

#### Experiment 1: Follicle recruitment following different durations of altrenogest treatment, subsequent superstimulation with varying eCG doses, and the use of different flow rates

**Fig. 1:**
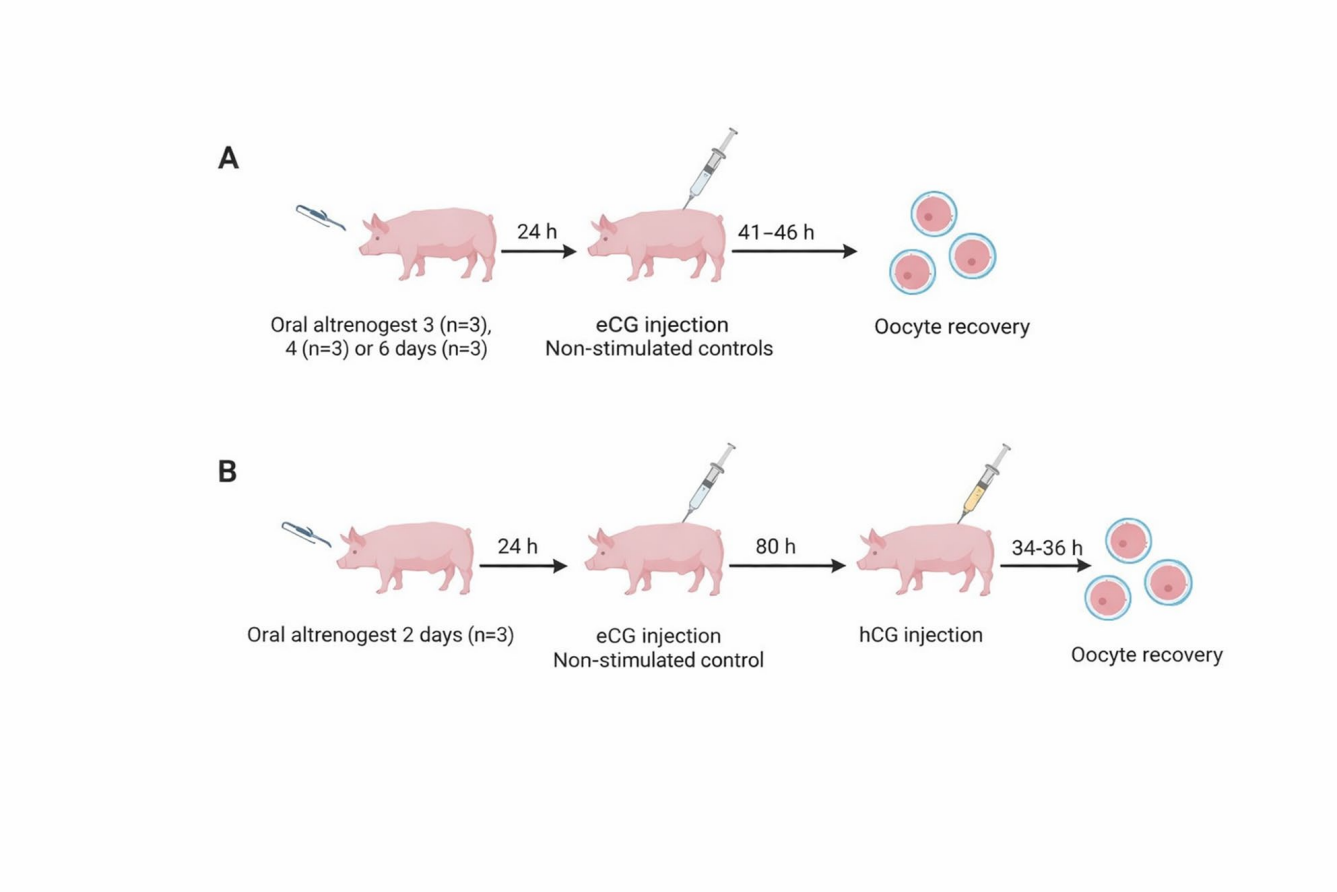
Treatments administered to the 12 sows in Experiment 1

The Experiment involved 12 animals that were block randomized to treatments in groups of three. Here, oestrus was postponed by administration of altrenogest to schedule OPU procedures on consecutive weekdays. The same protocol was used to obtain in vivo matured oocytes. The animals were administered 20 mg/day of altrenogest (Altresyn 4 mg/mL, Ceva, Loudéac, France) orally at 3 pm for 2, 3, 4 or 6 days respectively, starting on the day before weaning. Groups of three animals were randomly assigned so that, 24 h (h) after the final altrenogest treatment, one sow received 1000 IU eCG (Folligon, Intervet International B.V., AN Boxmeer, The Netherlands) intramuscularly, another received 1500 IU eCG, and the third served as a non-stimulated control. For the eCG-treated sows, OPU was performed 41–46 h after eCG administration, while the control sow underwent OPU at the corresponding time point (Fig. 1A). The three animals receiving two days of altrenogest treatment were also administered eCG 24 h after the final altrenogest dose. Additionally, all animals received 500 IU hCG (Chorulon vet., Intervet International B.V., Boxmeer, The Netherlands) intramuscularly 80 h after the last altrenogest dose to stimulate and synchronize the maturation of the oocytes. OPU was then conducted 34–36 h after hCG administration (Fig. 1B). During OPU, the two ovaries of each sow were punctured with flow rates of either 24 or 30 mL of water per minute. The ovaries were randomly assigned to each flow rate prior to the procedure.

#### Experiment 2: Effects of superstimulation with varying doses of eCG, day of OPU post-weaning and two different flow rates

**Fig. 2:**
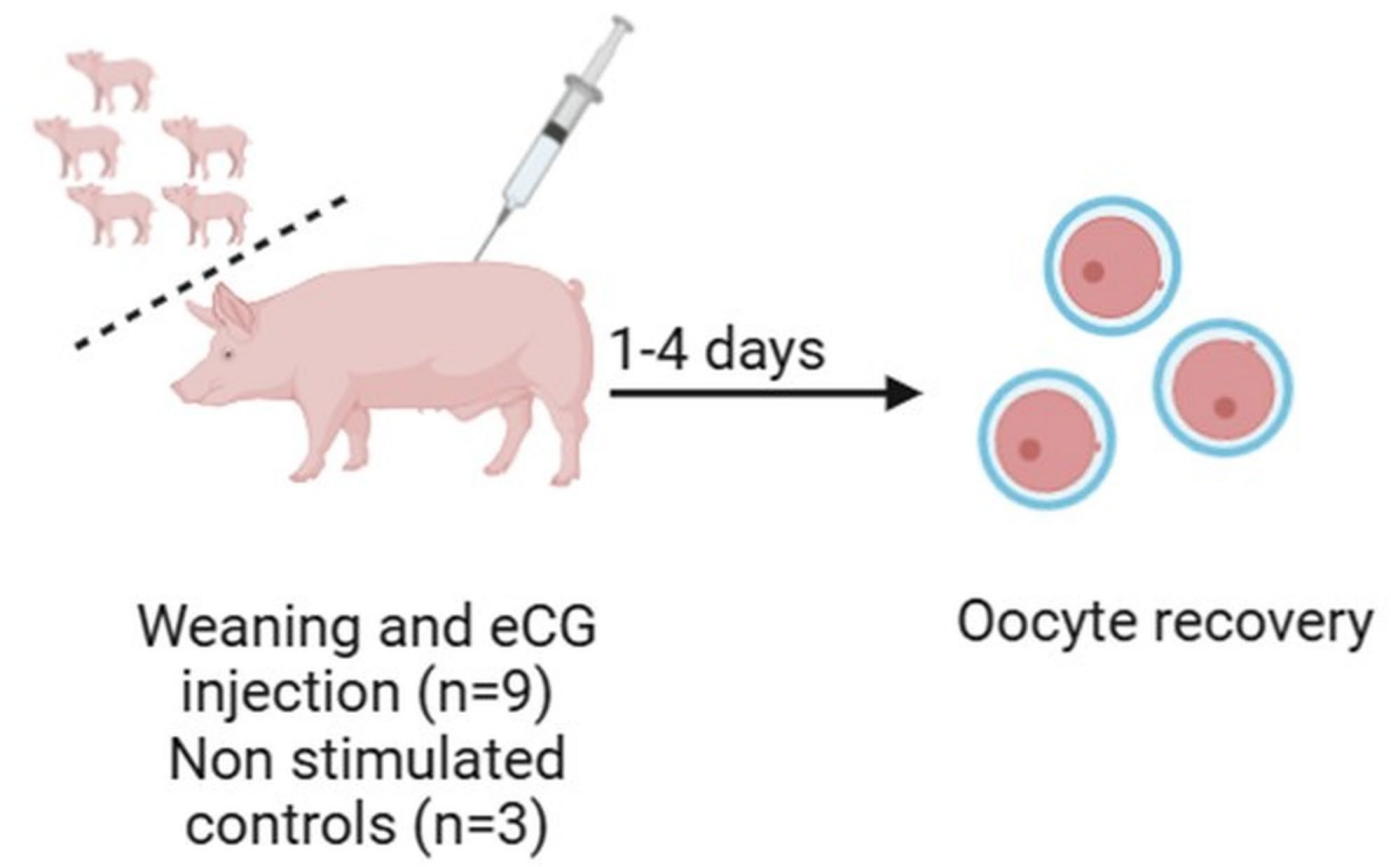
Treatments administered to the 12 sows in Experiment 2

Experiment 2 aimed to evaluate the effects of superstimulation using eCG, the timing of OPU post-weaning, and flow rates of either 24 or 30 mL of water per minute on oocyte retrieval. Twelve animals were enrolled at weaning and organized into blocks of three. OPU was conducted one, two, three, or four days after weaning, depending on the block within each block. One sow received 1000 IU eCG and another received 1500 IU eCG intramuscularly on the day of weaning; the third sow served as an unstimulated control (Fig. 2). Flow rates of either 24 or 30 mL of water per minute were used for puncture of either of the ovaries of the animals, followed by grading of the collected cumulus-oocyte complexes (COCs).

#### Experiment 3: Effect of partial in vivo maturation on embryo production

**Fig. 3:**
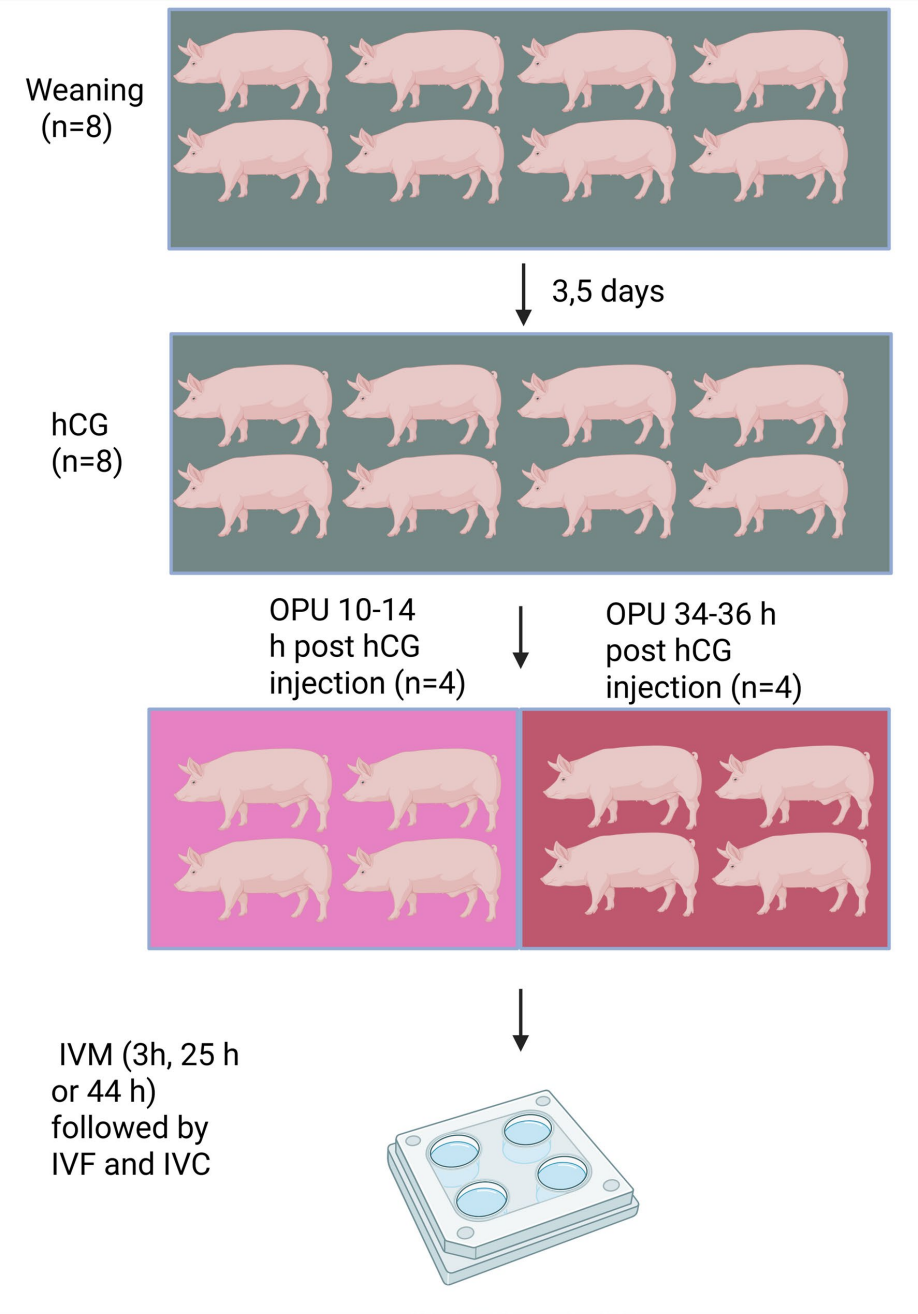
Methods for oocyte retrieval and in vitro embryo production in Experiment 3 (8 sows)

Experiment 3 included 8 animals. The animals were administered 500 IU of hCG i.m. 3,5 days post weaning. On four of the animals, OPU was performed 10–14 h post hCG treatment. On the other four animals, OPU was performed 34–36 h post hCG treatment (Fig. 3). All follicles were aspirated with a flow rate 30 mL of water per minute. All media used for in vitro embryo production were purchased from EmbryoCloud (Murcia, Spain); NaturARTs-PIG-IVM1-LYO (IVM1) and NaturARTs-PIG-IVM2-LYO (IVM2) were used for oocyte maturation (IVM), NaturARTs-PIG-IVF-LYO for fertilization (IVF), and Natur-ARTs-PIG-IVC2-LYO for in vitro culture (IVC).

The OPU-derived oocytes were washed in TL-HEPES-PVA and transferred to Eppendorf tubes containing 1 mL of the same media. Using a portable embryo transport incubator (Minitube, Ref.: 19180/0001), a stable temperature of 37 °C was ensured during the 30 min transport from the farm to the laboratory.

COCs collected 10–14 h after hCG administration were first washed once in a 300 µL droplet of IVM1 medium and subsequently cultured for 20 h in 500 µL IVM1 in a Nunc four-well dish at 38.5 °C under 5% CO₂ in a humidified atmosphere. After this initial culture period, the oocytes were washed twice in 300 µL droplets of IVM2 medium and transferred to 500 µL IVM2. Unexpanded oocytes (upon OPU) were matured for another 24 h (20 h in IVM1 followed by 24 h in IVM2, in total 44 h in vitro maturation). These oocytes were called fully in vitro matured oocytes. Expanded oocytes (upon OPU) were matured only for 5 h in IVM2. These oocytes were called partially in vivo matured oocytes (in total 25 h in vitro maturation). COCs collected 34–36 h post hCG were directly placed in IVM2 medium and cultured for 3 h prior to in vitro fertilization (in total 3 h in vitro maturation). These oocytes were also called partially in vivo matured oocytes.

Fresh extended semen (Norsvin, Hamar, Norway) was used for IVF and the semen was centrifuged with a two-layered BoviPure gradient (Nidacon, Mölndal, Sweden) to remove semen extender. Briefly, 2 mL of 40% BoviPure was layered on top of 2 mL 80% BoviPure in a 15 mL tube, after which 1 mL of extended semen was carefully layered on top. The semen was centrifuged for 25 min at 300 x g, after which the supernatant was removed. The pellet was resuspended in 300 µL BoviWash and 5 µL was diluted further with 145 µL IVF medium to get to a concentration of approximately 1 × 10^6^ motile sperm cells /mL.

During sperm centrifugation, the COCs were washed once in a droplet of 300 µL IVF medium and then moved to 470 µL IVF medium. After centrifugation and the final dilution step, 30 µL of the final sperm solution was added to each well. Oocytes were co-incubated with approximately 60,000 million motile sperm cells /mL (Ratio 1000:1). After 2 h of co-incubation, oocytes were transferred to a new well with 500 µL fresh IVF medium to remove an excess of sperm cells.

**Fig. 4:**
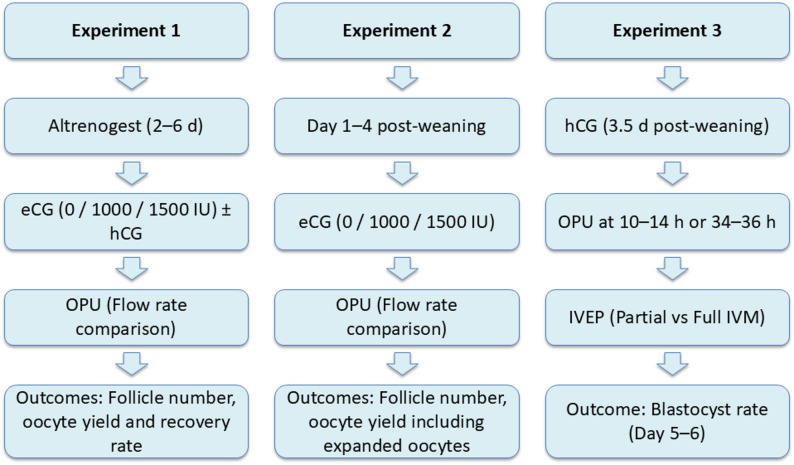
Representative cumulus–oocyte complexes (COCs) collected by transvaginal ovum pick-up (OPU) and the resulting embryos after in vitro embryo production (IVEP). A) COCs collected 10–14 h after hCG administration. B) Expanded COCs collected 10–14 h after hCG administration, representing partial in vivo maturation. C) Expanded COCs collected 34–36 h after hCG administration, representing more advanced in vivo maturation

After a total of 4 h co-incubation, presumptive zygotes were denuded of cumulus cells by pipetting them 30 times. They were washed twice in a 300 µL droplet with TL-HEPES-PVA [29] and once in a droplet with IVC medium before culture in 500 µL IVC medium under 400 µL mineral oil (IVF Biosciences, Falmouth, UK) at 38.9 °C in an humified atmosphere containing 6% CO_2_ and 7% O_2_. Blastocyst rates were assessed on day 5 of culture. The blastocyst rate was defined as the number of blastocysts divided by the total number of fertilized and cultured oocytes. An embryo with a clear blastocoel was defined as a blastocyst. A schematic overview of the Experiments 1, 2 and 3 is presented in Fig. 4.

### Statistical analysis

Statistical analyses were performed using JMP^®^ Pro version 17.2 (SAS Institute Inc., Cary, NC, USA). Analysis of variance (ANOVA) was utilized to evaluate differences between various treatments, considering only main effects and excluding interaction effects. The response variables fulfilled the test assumptions of, independence, homogeneity of variance, linearity, and normality of residuals. The Shapiro-Wilk test was used to verify normality, with non-significant results indicating that the data met this assumption. The other two factors, homogeneity of variance and linearity, were assessed visually. All analyses were conducted in JMP, with the significance level set at 0.05. Descriptive data are presented as arithmetic means ± standard deviation.

## Results

### Sedation protocol (Experiments 1 to 3)

All sows reached a level of sedation characterized by depressed consciousness and tolerance of rectal palpation and OPU without marked signs of distress. The procedure was completed as planned for all animals. Based on operator assessment, the inclusion of sedation and epidural anaesthesia provided improved procedural stability and handling conditions compared with our previously reported physically restrained OPU without pharmacological immobilization. The inclusion of sedation and epidural anaesthesia resulted in a more controlled and predictable procedural workflow. No significant adverse events related to sedation or epidural anaesthesia were observed.

### Oocyte retrieval overview (Experiments 1 to 3)

There was a significant difference between the Experiments (Table 1) in both number of recovered oocytes (F_(2,29)_ = 16.00; *P* = < 0.01), follicles aspirated (F_(2,29)_ = 14.90; *P* = < 0.01), and recovery rates, i.e., percentage of number of recovered oocytes compared to number of aspirated follicles (F_(2,26)_ = 5.61; *P* = < 0.01).

**Table 1:** Overview of results from Experiments 1 to 3

Sows (*N*)	Exp 1	Exp 2	Exp 3	*P*
	12	12	8	
Average OPU duration (min)	23.0	26.6	24.9	0.34
Aspirated follicles (avg)	27.8^a^	54.8^b^	21.8^a^	< 0.01
Recovered oocytes (avg)	7.9^a^	30.3^b^	12.8^a^	< 0.01
Recovery rate (%, avg)	27.3%^a^	55.3%^b^	51.8%^b^	< 0.01

Different superscript letters (a, b) within rows indicate significant differences (*P* < 0.05). Recovery rate is calculated as the percentage of recovered oocytes relative to aspirated follicles. Values are presented as mean ± standard deviation

#### Effects of follicle selection with varying length of altrenogest and subsequent superstimulation with varying doses of eCG (Experiment 1)

In the two blocks of animals in Experiment 1 receiving altrenogest for 2 or 6 d, animals that received eCG (sow ID 8, 9, 11 and 12) had less than five follicles > 4 mm (Table 2), possibly due to early ovulation. Excluding these animals, we recovered on average 11.4 ± 4.9 oocytes per session from 38.8 ± 6.9 follicles, resulting in a recovery rate of 29.4%. Small follicles were aspirated from sows ID 11 and 12 but yielded only 2 oocytes per sow. The sow with the highest number of aspirated follicles and recovered oocytes received altrenogest for three d and were superstimulated with 1500 IU eCG.

**Table 2:** Retrieved oocytes in Experiment 1

Sow no.	Parity	Treatment	Follicles	Oocytes	Grade E	Retrieval rate
1	5	ALT3-CON	36	6	0	17%
2	5	ALT3-1000	38	12	0	32%
3	5	ALT3-1500	54	21	0	39%
4	5	ALT4-CON	37	8	1	22%
5	5	ALT4-1000	36	9	0	25%
6	6	ALT4-1500	36	9	0	25%
7	5	ALT2-CON-h	42	16	4	38%
8	5	ALT2-1000-h	0	0		
9	7	ALT2-1500-h	0	0		
10	5	ALT6-CON	31	10	0	32%
11	5	ALT6-1000	6	2	0	33%
12	6	ALT6-1500	18	2	0	11%

Sows were administered altrenogest (ALT) for a period ranging from 2 to 6 days (ALT2, ALT3, ALT4 or ALT6). Following the final dose of altrenogest, two animals in each block were injected with either 1000IU or 1500IU of eCG, with the remaining sow serving as a non-stimulated control (CON). OPU was carried out two days post eCG administration in three blocks of animals. In the block with animals 7–9, hCG (h) was administered 80 h after eCG, and OPU was conducted 34–36 h following the hCG administration

We found similar recovery rates and oocyte qualities between the two flow rate settings in Experiment 1 (Table 3). A total of 45 and 37 oocytes were retrieved with flow rates of 24 and 30 mL of water per minute during Experiment 1, respectively.

**Table 3:** Retrieval rates (RR) and oocyte quality in Experiment 1 using flow rates of 24 and 30 mL of water per minute

	40 mmHg	60 mmHg
Aspirated follicles	154	182
Recovered oocytes	45	37
Retrieval rates (RR)	29%	20%
A	33%	22%
B	13%	8%
C	27%	41%
D	24%	30%
E	2%	0%

Recovery rate is calculated as the percentage of recovered oocytes relative to the number of aspirated follicles. Oocyte quality is categorized into grades A–E based on the morphological appearance of the cumulus-oocyte complex

### Effect of day of the follicular phase, superstimulation by eCG and flow rate (Experiment 2)

We recovered on average 30.3 ± 11.0 oocytes per session from 54.8 ± 10.6 follicles, resulting in a recovery rate of 55.3% (Table 4). The number of aspirated follicles (F_(3,8)_ = 2.50; *P* = 0.13) and retrieved oocytes (F_(3,8)_ = 1.95; *P* = 0.20) did not significantly differ between the various days post weaning. Additionally, the administration of eCG did not lead to any significant differences in the number of aspirated follicles (F_(2,9)_ = 1.07; *P* = 0.38) or retrieved oocytes (F_(2,9)_ = 1.95; *P* = 0.20). Notably, 83% of the oocytes retrieved on day 4 post weaning were surrounded by expanded cumulus cells. Grade E oocytes were otherwise found only in superstimulated sows. We found similar recovery rates and oocyte qualities when comparing the flow rates of 24 and 30 mL of water per minute (Table 5).

**Table 4:** Data from individual sows in Experiment 2 showing the effect of superstimulation and day post-weaning on the number of aspirated follicles, total number of oocytes retrieved, and the number of oocytes surrounded by expanded cumulus cells (Grade E)

Sow no.	Parity	Day post-weaning	eCG	Follicles	Oocytes	Grade E	Recovery rate
13	5	1	0	56	22	0	39%
14	5	1	1000	36	23	4	64%
15	5	1	1500	38	11	7	29%
16	5	2	0	66	48	0	73%
17	5	2	1000	55	29	1	53%
18	5	2	1500	59	35	0	59%
19	5	3	0	52	48	0	92%
20	5	3	1000	59	26	1	44%
21	5	3	1500	65	17	0	26%
22	5	4	0	62	37	28	60%
23	6	4	1000	46	29	23	63%
24	5	4	1500	63	38	27	60%

Recovery rate is calculated as the percentage of oocytes retrieved relative to the number of aspirated follicles

**Table 5:** Recovery rates (RR) and oocyte quality in Experiment 2 using flow rates of 24 and 30 mL water perminute

	24 mL water/ min	30 mL water/ min
Aspirated follicles	334	323
Recovered oocytes	179	184
Recovery rates (avg)	54%	57%
A	15%	13%
B	9%	11%
C	23%	22%
D	26%	31%
E	27%	23%

Recovery rate is calculated as the percentage of recovered oocytes relative to the number of aspirated follicles. Oocyte quality is categorized into grades A–E based on the morphological appearance of the cumulus-oocyte complex

### Effect of partial in vivo maturation on embryo production efficiency (Experiment 3)

There was a major variation in the oocyte retrieval outcomes in this Experiment (Table 6).

**Table 6:** Data from individual sows in Experiment 3, detailing the number of aspirated follicles, retrieved oocytes, and oocytes with expanded cumulus oophorus (Grade E), indicating the start of in vivo oocyte maturation

Sow no.	Parity	Day post-weaning	Follicles	Oocytes	Grade E	Recovery rate
25	5	4	45	24	0	53%
26	5	4	16	8	0	50%
27	5	4	30	30	26	100%
28	5	4	32	10	0	31%
29	5	5	0	0	0	
30	6	5	38	26	26	68%
31	5	5	7	3	1	43%
32	5	5	6	1	1	17%

**Fig. 5:**
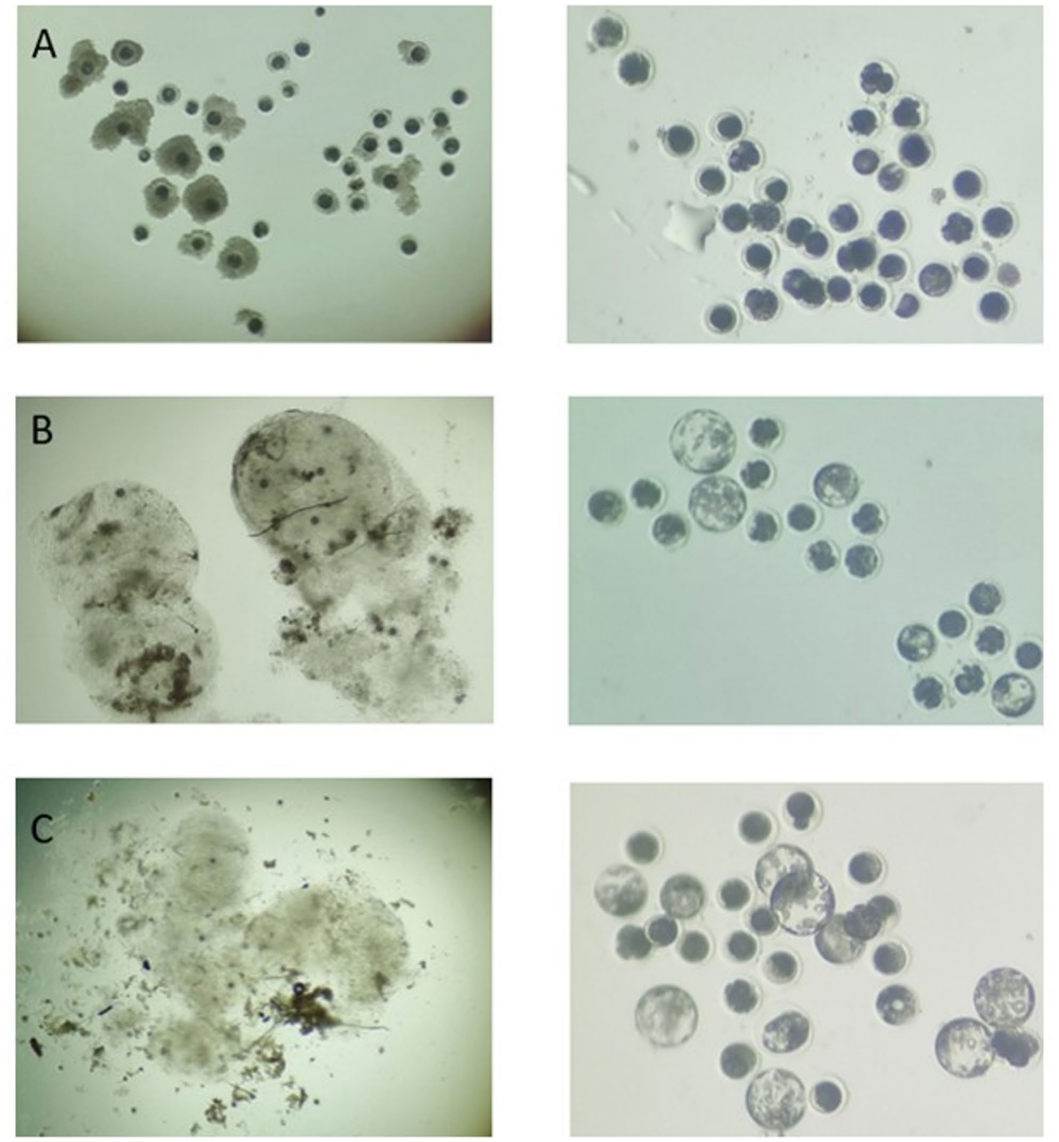
Oocytes collected by OPU (left column) and day 5 embryos produced (right column). (**A**) COCs collected 10–14 h post hCG administration. (**B**) Expanded COCs collected 10–14 h post hCG administration. (**C**) Expanded COCs collected 34–36 h post hCG administration

Following aspiration 10–14 h post hCG, 18.0 ± 9.3 COCs were recovered per sow, with 52.9% of these having expanded cumulus cells. In the animals subjected to OPU 34–36 h post hCG, only one sow had mature follicles on the ovaries. This animal yielded 26 oocytes, of which all were surrounded by expanded cumulus cells. A higher blastocyst rate was obtained for the partially in vivo matured oocytes (25 h–3 h IVM) compared to in vitro matured oocytes (44 h IVM) (Table 7). Comparable blastocyst rates (33%) were obtained for expanded COCs collected 10–14 h post-hCG and matured for 25 h IVM (7/21), and for those collected 34–36 h post-hCG with only 3 h of maturation (10/30) (Fig. 5).

**Table 7:** Oocytes collected and blastocysts rates in Experiment 3

Hours after hCG	Hours of IVM	Oocytes collected	Blastocyst rate (D5 or D6)
10–14	44	Immature COCs	6% (2/36) on D5
10–14	25	Expanded in vivo matured COCs	33% (7/21) on D6
34–36	3	Expanded in vivo matured COCs	33% (10/30) on D6

## Discussion

We achieved our primary goal of enhancing the efficiency of oocyte retrieval in multiparous sows through transvaginal OPU by incorporating anaesthetics and analgesics into our already established protocol [10]. In our previous work, oocyte recovery was performed in physically restrained, non-sedated sows, which proved technically feasible but was associated with animal movements and increased procedural difficulty, and average recovery rates of 7.7 and 9.0 oocytes per session were achieved [10, 11]. The implemented anaesthetics and analgesics protocol successfully enabled the retrieval of numerous oocytes, with Experiment 2 yielding an average of 30.3 oocytes per sow, which is considerably higher than the numbers described in previous studies [8–12, 16]. Recovery rates of 48 oocytes from two individual animals were achieved.

Sedation and epidural anaesthesia can increase the feasibility of transvaginal OPU in commercial settings by improving immobilization and facilitating transrectal manipulation of the ovaries, which may reduce procedure-related stress and allow more consistent follicle puncture. However, these measures also increase complexity, as they require trained personnel, appropriate handling facilities, drug availability, and time for preparation and post-procedural monitoring. In addition, the practical feasibility will depend on the duration of each session, the number of animals that can be handled per day, and farm-specific logistics.

With respect to potential OPU repetition frequency and recovery time, evidence in sows remains limited. In the present study, meloxicam was administered for post-procedural analgesia, and the applied epidural dose was selected to reduce discomfort while aiming to maintain hindlimb function. Under field conditions, recovery is expected to be rapid, but systematic welfare assessment was not performed and should be addressed in future controlled studies. Observations from our previous work indicate that repeated OPU sessions can be performed across consecutive oestrus cycles. In one study, three sows underwent OPU during 3, 4, and 6 consecutive cycles under general anaesthesia, respectively. The sows subjected to three and four OPU sessions were examined postmortem at the conclusion of the study. No macroscopic lesions were observed that would suggest impaired reproductive function. Following six OPU sessions, the third sow was inseminated and later farrowed 16 live-born piglets [11]. However, given the current evidence base, a conservative approach in commercial practice with regards to repeating OPU in the same sow is recommendable, until larger studies systematically evaluate both short- and long-term welfare outcomes (e.g., pain-related behaviours, locomotion, feed intake, reproductive tract health, and subsequent fertility). Further research should also determine minimum recovery intervals and assess potential cumulative effects when procedures are repeated across multiple cycles.

In these Experiments, we added epidural anaesthesia to the protocol, using the easily accessible lumbosacral space for administration. Since the blocking of the nerve roots by injection in the lumbosacral space can interfere with the innervation of the hind limbs [30], we had to limit the quantity of procaine used to avoid causing the sow to become recumbent inside the chute following the procedure. During a pilot test prior to this Experiment, we observed animals with slight hind leg paresis already at a dose of 25 mg, and thus we decided to use 20 mg (data not published). The epidural injection was facilitated by the animals’ position inside the chute and conveniently applied. Although not evaluated in the current study, we think that the inclusion of epidural anaesthesia helped reduce intestinal straining during the procedure. Though a higher dose might potentially have enhanced this effect even further, there was a risk of long-lasting recumbency, not compatible with proper animal welfare due to the design of the restraint chute. If a system could continue operating even if the sow remains recumbent under anaesthesia until the epidural effect wears off, it will allow for administration of higher doses of anaesthesia and potentially even more precise manipulation of the ovaries and complete pain relief. Since the inclusion of sedation and epidural anaesthesia in this study appeared to improve handling conditions and procedural stability, and thereby allowing a more controlled follicular aspiration, further refinement of pharmacological and physical restraint protocols in porcine OPU is warranted. By modifying the OPU device and including a metal cone at the tip of the needle guide, we had the impression that the puncturing precision of follicles was improved. This may have contributed to higher recovery rates during these Experiments compared to our previous studies [10, 11], and certainly warrants further investigation and improvement.

We could not detect a significant difference in oocyte retrieval outcomes between the eCG-stimulated animals and controls in Experiment 2. The limited sample size in this study may have reduced the statistical power to detect significant differences between the treatment groups. The low sample size was due to the nature of the field study and the availability of animals at the sow pool. Initially, the first protocol (Experiment 1) was intended to serve as the setup for the full Experiment but proved ineffective. Consequently, adjustments were made to enhance efficiency (Experiments 2 and 3).

One could argue that treating sows with eCG for superovulation purposes will not provide additional practical benefit. After all they are poly-ovulatory animals. In addition, excessive stimulation may alter the follicular environment and potentially affect oocyte quality, as described in other species [31–33]. Exogenous gonadotropin treatment, particularly eCG due to its prolonged FSH- and LH-like activity, may theoretically disrupt physiological follicular dynamics if not optimally timed. Such disruption could increase the likelihood of persistent or luteinized follicles. However, the occurrence of cystic ovarian structures following short-term superstimulation in sows remains insufficiently documented. Given that sows already produce large numbers of ovulatory follicles, routine superstimulation may not be necessary under all conditions. A targeted approach, reserving superstimulation for specific high-value donors or experimental settings, may represent a more balanced strategy.

In this study the superovulation and synchronization protocol was chosen since eCG may increase the number of ovulatory follicles [24]. Oocytes that were aspirated on the first day post-weaning from eCG-superstimulated sows were frequently surrounded by expanded cumulus cells, which may reflect the LH-like activity of eCG. Furthermore, in sows where oestrus was postponed for six days using altrenogest initiated the day before weaning, ovulation may have occurred as early as two days after eCG administration, since only few and small follicles were visible by ultrasound examination. A previous report showed that seven days of altrenogest treatment beginning the day before weaning allowed continued follicular growth, with follicles reaching an average diameter of 4.8 mm at the end of treatment [34]. Prolonged follicular growth under sustained progestagen exposure may alter follicular dynamics and oocyte competence. In cattle, extended dominance of preovulatory follicles has been associated with reduced developmental potential of the oocyte and impaired fertility [35]. Here, the continued growth of follicles under altrenogest treatment might have led to a population of medium-sized follicles expressing LH-receptors when eCG was administered. These follicles could then respond to the LH effect of eCG by ovulating. Our poor results with altrenogest in Experiment 1 align with the findings of Gonzalez-Ramiro et al., which indicate that short-term altrenogest treatments combined with superstimulation may lead to the release of a large number of immature oocytes and negatively affect pregnancy and embryo production efficiency [36].

As mentioned in the Background section, the LH surge is important for inducing cumulus expansion and germinal vesicle breakdown [21]. Using hCG to simulate this surge for synchronizing follicle maturation might prove beneficial in ensuring a homogenous pool of oocytes prior to OPU. This would be particularly important if the protocol were to depend on partial in vivo maturation. In such scenarios, it would be essential to ensure a minimal variation in terms of oocyte maturation timing. Brussow [23] recommends that for hormonal synchronization of ovulation of sows weaned after a lactation length of more than 4 weeks, hCG should be given 56–58 h after the eCG injection, which should be administered 24 h post-weaning. Shortening of the interval between eCG and hCG seems necessary for future experiments utilizing fixed time synchronization. In Experiment 1, 50% of the superstimulated animals had probably ovulated before the OPU procedure. In Experiment 3, we found that by day five post weaning in naturally cycling animals, 5 out of 8 sows had probably already ovulated. Therefore, future studies should include a comprehensive evaluation of stimulation protocols and careful monitoring of ovarian responses in sows.

We were able to consistently recover oocytes during the first four days of the oestrus cycle. This aligns with previous research suggesting that multiple follicles are available at weaning in modern hyperprolific sows [13, 14]. Given that these oocytes from sows possess higher developmental competence than oocytes obtained from ovaries originating from prepubertal gilts at the abattoir, this technique could be valuable for future OPU-IVEP procedures. Sourcing oocytes from monitored specific pathogen-free farms will also reduce potential risks associated with disease transmission compared to collecting oocytes from ovaries obtained at slaughterhouses.

During the aspiration process, complete avoidance of blood contamination during follicular aspiration proved challenging. Therefore, it was necessary to quickly flush the needle and tubes to avoid clot formation and potential loss of oocytes within these clots. Moreover, the time the oocytes remained in the collection tube exposed them to environmental temperatures, potentially leading to cooling that could compromise their developmental competence. We could not find major differences in oocyte quality when comparing flow rates of 24 and 30 mL of water per minute. As a result, we chose to apply the flow rate of 30 mL of water per minute for oocyte retrieval in Experiment 3.

We observed high recovery rates from many sows that were close to the timepoint of ovulation. Our experience aligns with findings from laparoscopic OPU in gilts, where 80.8% of the follicles aspirated 34 h after hCG administration yielded oocytes, compared to only 48.5% of the follicles aspirated 2 h prior to hCG administration [37]. This could potentially be due to the expansion of the cumulus oophorus following the LH surge, which increases the hyaluronic acid content in the extracellular matrix, thereby weakening the attachment to the basal lamina [38]. Another notable benefit with aspirating expanded COCs was that a major proportion of these oocytes remained covered by cumulus cells following aspiration. This suggests that allowing the cumulus to expand before aspiration could result in fewer oocytes being denuded of cumulus cells, which is known to be detrimental to their developmental competence [18].

Incomplete cytoplasmic maturation of the oocyte cause challenges with porcine in vitro embryo production [39, 40]. This could be circumvented by maturing the oocytes in vivo, potentially leading to improved efficiency in porcine embryo production. The advantages of in vivo maturation have been highlighted by previous experiments in pigs [20], as well as in cattle [41] and humans [4]. The duration of in vitro maturation in our Experiment was chosen based on the expected time before the predicted ovulation. Blastocyst rates of 33% from the expanded oocytes retrieved in Experiment 3 indicates the potential of increasing oocyte quality by maturation in vivo. Optimization of this protocol may further improve these results and lead to consistent high efficiency in porcine in vitro embryo production.

## Data Availability

The datasets from the current study are available from the corresponding author on reasonable request.
